# Graceful Giving

**Published:** 1914-07-11

**Authors:** Henrietta O. Barnett

**Affiliations:** Hampstead Garden Suburb, N.W.


*To the Editor of* The Hospital.

### Graceful Giving.

*To the Editor of* The Hospital.

Sir,—Many years ago Canon Barnett and I were staying at Bordighera, so as to be near Mr. George Macdonald and breathe the atmosphere of his moral heights. One evening at the hotel a printed appeal to subscribe to some charity was given to each visitor. Most people looked bored, or antagonistic, or assumed that expression of sage disapproval which passes for wisdom, but one lady, Miss Jeannie Ridley, was overheard to say to her sister, "Annie, here is an opportunity for helping, we must look into it and see what we can do."

I write at the request of the executive of the Children's Country Holidays Fund (18 Buckingham Street, Strand, W.C.), who wish your readers to know that they desire to send this summer 50,000 children to stay for two weeks in the families of country cottagers, there to have fun and freedom, gaiety and gladness, health and happiness. To give this amount of joy will cost £25,000, or 10s. a child, for the parents practically pay fares, &c., and the management is mainly voluntary.

In 1891 my husband and I were visiting Miss Jane Addams in Chicago, where I was to have the great pleasure of opening her Loan Art Gallery, erected in imitation of ours in Whitechapel. She was then busy building up her giant organisation for social helpfulness, and money was much needed. One day she received a cheque for a large sum, but quietly returning it to its envelope she said, "That must go back, it's tainted money." "Go back! Why?" was asked. "People must be shown it is a privilege to be allowed to give, and that I accept only clean gains for service money" she replied.

Across the continents, across the years, two women struck two notes of a chord in the national anthem of charitable progress.

Will those who have "clean" money, and "count it a privilege to give," use the "opportunity of helping" which the Children's Country Holidays Fund provides by sending 50,000 out of London's 800,000 poor children to obtain gains which, after all, are their birthrights, a knowledge of Nature, an experience of pure pleasure, and a better chance of health?—I am, Sir, yours truly,

Henrietta O. Barnett.

Hampstead Garden Suburb, N.W.,
June 26, 1914.

